# Key Node Identification Method Based on Multilayer Neighbor Node Gravity and Information Entropy

**DOI:** 10.3390/e26121041

**Published:** 2024-11-30

**Authors:** Lidong Fu, Xin Ma, Zengfa Dou, Yun Bai, Xi Zhao

**Affiliations:** 1 College of Computer Science and Technology, Xi’an University of Science and Technology, Xi’an 710064, China; fulidong2005@163.com (L.F.); maxin067@163.com (X.M.); baiyuncarrie@xust.edu.cn (Y.B.); 2 School of Computer and Information Science, Qinhai Institute of Technology, Xining 810016, China; 3 Xi’an Big Data Service Center, Xi’an 710064, China; zhaoxi2024@gmail.com

**Keywords:** complex networks, key nodes, inter-node gravity, information entropy

## Abstract

In the field of complex network analysis, accurately identifying key nodes is crucial for understanding and controlling information propagation. Although several local centrality methods have been proposed, their accuracy may be compromised if interactions between nodes and their neighbors are not fully considered. To address this issue, this paper proposes a key node identification method based on multilayer neighbor node gravity and information entropy (MNNGE). The method works as follows: First, the relative gravity of the nodes is calculated based on their weights. Second, the direct gravity of the nodes is calculated by considering the attributes of neighboring nodes, thus capturing interactions within local triangular structures. Finally, the centrality of the nodes is obtained by aggregating the relative and direct gravity of multilayer neighbor nodes using information entropy. To validate the effectiveness of the MNNGE method, we conducted experiments on various real-world network datasets, using evaluation metrics such as the susceptible-infected-recovered (SIR) model, Kendall τ correlation coefficient, Jaccard similarity coefficient, monotonicity, and complementary cumulative distribution function. Our results demonstrate that MNNGE can identify key nodes more accurately than other methods, without requiring parameter settings, and is suitable for large-scale complex networks.

## 1. Introduction

As research into complex networks deepens, researchers are paying increasing attention to areas like link prediction [1], community detection [2], and key node identification [3]. In complex networks, nodes represent individuals, and edges represent the connections between them. By representing entities as nodes and their relationships as edges, many complex systems in the real world, such as social networks, biological networks, disease networks, and transportation networks, can be modeled as complex networks. Identifying key nodes within these networks has become a prominent research focus due to their critical role in controlling information dissemination [4]. For example, in social networks, identifying key nodes can effectively curb the spread of misinformation and harmful public opinion. In biological networks, identifying key nodes can help discover potential drug targets to design specialized therapeutic methods. In disease networks, the key nodes are individuals with a high risk of spreading, and identifying these nodes can effectively stop the spread of infectious diseases. Similarly, in transportation networks, the key nodes are usually important traffic hubs, and identifying them can help optimize traffic flow and prevent traffic accidents.

Many communication phenomena in the real world, such as the diffusion of information or the spread of diseases, can be abstracted as communication processes on complex networks. There is a close and interactive connection between these propagation processes and the structure of the network. In order to effectively analyze and control the propagation of information in complex systems, Zenil et al. [5] proposed an approach based on algorithmic information theory designed to control and reprogram complex systems in dynamic spaces. The method is able to deeply analyze the dynamic propagation characteristics of the nodes in the network and accurately identify the key nodes that play a decisive role in the information propagation process by evaluating the algorithmic information content of the nodes.

Currently, numerous scholars have proposed various methods to identify key nodes in complex networks. For example, degree centrality (DC) [6], which evaluates a node based on the number of its neighboring nodes, is simple to compute but generally has low accuracy. Closeness centrality (CC) [7] offers higher accuracy than degree centrality but cannot handle networks with disconnected nodes. Betweenness centrality (BC) [8] measures a node’s importance by assessing its role as a ‘bridge’ in the network; however, if a node is not on the shortest path between any pair of nodes, its BC value will be zero, making it impossible to evaluate the node’s influence. Eigenvector centrality (EC) [9] considers not only a node’s direct connections but also the importance of its neighboring nodes. PageRank (PR) [10] and LeaderRank (LR) [11] further refine this by considering that a node’s influence depends not only on the number of its neighboring nodes but also on the contribution of those neighboring nodes. In addition, Kitsak et al. [12] proposed the K-shell decomposition (K-shell) method, which determines a node’s importance based on its position in the network by assigning each node a K-shell index, with nodes closer to the core of the network being assigned a higher K-shell index. While K-shell decomposition improves upon previous methods in terms of accurately assessing node influence, it lacks monotonicity and cannot further distinguish between nodes with the same K-shell index. To address this issue, Bae and Kim [13] proposed the Extended Neighborhood Coreness Centrality (CNc+) method, which evaluates a node’s importance by summing the K-shell values of its neighboring nodes. Namtirtha et al. [14] further introduced the Weighted K-shell Degree Neighborhood (KSD^W^) method, which integrates a node’s degree, K-shell index, and the information from its neighboring nodes to provide a more comprehensive assessment of the node’s influence in the network. Zhao et al. [15] combined the K-shell method with the Structural Hole (SH) theory, proposing the SHKS method to measure a node’s importance by considering both its network position and its role in bridging structural gaps.

Each of the above methods has its strengths in identifying key nodes, with most focusing on the density of connections and positional advantages, considering nodes that are more closely connected to their neighbors to be more important. However, both the KSD^W^ and SHKS methods depend on parameter settings for identifying key nodes, and varying these parameters can influence the accuracy of results. Additionally, overlooking the interaction between nodes and their neighbors when assessing node influence can further affect the precision of key node identification. Based on the above considerations, we propose a key node identification method based on multilayer neighbor node gravity and information entropy (MNNGE). This method quantifies the interactions between nodes by calculating the relative and direct gravitational forces between them and then determines node centrality by aggregating these forces across multiple layers of neighboring nodes using information entropy. In order to evaluate the performance of the MNNGE method, we compared its effectiveness with other methods using the SIR model, Kendall τ correlation coefficient, Jaccard similarity coefficient, monotonicity, and the complementary cumulative distribution function on nine real-world network datasets. The experimental results show that the MNNGE method can accurately and effectively identify key nodes.

## 2. Related Work

### 2.1. Research and Development of Key Node Identification Methods

In this section, the main work related to identifying key nodes in complex networks is reviewed.

Degree centrality [6] is one of the most commonly used methods for identifying key nodes, which mainly assesses the importance of a node by calculating its number of direct connections, but it does not consider the global topology of the network or the interactions between neighboring nodes. Chen et al. [16] proposed a semi-local centrality method based on this approach, which takes into account the influence of a node’s fourth-order neighbors. While this method provides a more comprehensive evaluation, its computational complexity increases as the network size grows. The K-shell method [12] determines a node’s importance based on its position in the network; it considers nodes at the center of the network to have higher influence, and the method has low time complexity. Based on this, researchers have developed several improved methods, such as MDD [17], KSIF [18], and KSGC [19]. MDD method evaluates a node’s importance by considering the number of connections of both the removed node and the remaining nodes. The KSIF method estimates the node’s influence by utilizing its K-shell decomposition value and incorporating iterative information. The KSGC method is an enhanced gravity model based on K-shell, which takes into account the positional information of nodes to improve the accuracy of key node identification.

In recent years, the concept of entropy has been incorporated into node centrality analysis to enhance the identification of key nodes. For example, the ERM method [20] calculates the entropy centrality of a node’s neighbors and second-order neighbors using an information entropy approach to measure its spreading ability. The IKS method [21] combines the node’s K-shell value with an information entropy approach to identify key nodes. The SEGM method [22] integrates a node’s local influence, information entropy, and the gravity model, proposing a gravity model based on the entropy of the node to identify key nodes. In order to more comprehensively integrate the local and global information of nodes, Wu et al. [23] proposed a K-orders entropy-based method, which identifies key nodes in complex networks by comprehensively evaluating not only the entropy of different orders of a node’s nearest neighbor information but also the entropy of its betweenness centrality. Lu et al. [24] assessed the self-importance of nodes based on local structural entropy while using a network embedding algorithm to assess the global importance of nodes for a more comprehensive identification of key nodes. Compared with the classical methods, these methods have significantly improved their accuracy in identifying key nodes by introducing the concept of entropy centrality.

As research has deepened, scholars have found that applying neighborhood rules can improve the accuracy of identifying key nodes. For example, DAI et al. [25] proposed a method based on eigenvector centrality and local neighbor contributions, identifying key nodes by defining the node’s own influence and further calculating the contribution of its neighbors and the neighbors of those neighbors. Ullah et al. [26] combined degree, K-shell, the distance between nodes, and neighbor influence to identify key nodes. Zareie et al. [27], based on the clustering ranking method, considered the common hierarchical structure of nodes and their neighborhood influence. These studies show that methods applying neighborhood rules are more effective than those that do not when identifying key nodes.

### 2.2. Centrality Methods

**Definition 1.**  *Degree Centrality.*

*Degree centrality* [6] represents the number of neighboring nodes connected to a given node in the network. Let *k_i_* represent the degree centrality of node *i*, which is defined as

(1) $$k\left(i\right)=\sum_{j=1}^{N}a_{ij}$$

Where *N* is the total number of nodes, and *a_ij_* is the adjacency matrix of the network. If there is an edge between nodes *i* and *j*, *a_ij_* = 1, otherwise, *a_ij_* = 0.

**Definition 2.**  *K-shell Method*

The *K-shell* method [12] determines the importance of a node based on its position in the network and assigns each node in the network a *K-shell* index (*ks*-value), which indicates the topological position of the node in the network. In this method, nodes with degree 1 and their connecting edges in the network graph *G* are continuously deleted until no more such nodes appear in the network graph, at this stage all the deleted nodes are categorized into *1-shell* layer and assigned a *Ks* value of 1. Next, nodes with degree 2 and their connecting edges in the network are deleted, and these nodes are assigned a *Ks* value of 2. This process is repeated until all nodes in the network are assigned a *Ks* value. In this case, the node with the largest *Ks* value is the core node of the network, while the node with the smallest *Ks* value is the peripheral node. Typically, the node located in the core layer of the network has the greatest influence.

### 2.3. Information Entropy

The American scholar Shannon introduced entropy into information theory and proposed the concept of information entropy, which is used to quantify the amount of information contained in an event. In information theory, higher entropy indicates that the event contains more information. Specifically, if there is a random variable *X*, which may take the values $X=\left(x_{1},x_{2},\cdot \cdot \cdot ,x_{n}\right)$, and the probability that each value $x_{i}$ occurs is $p_{i}$ then the entropy of the random variable *X* is defined as

(2) $$E\left(x\right)=-\sum_{i=1}^{n}p_{i}*\log\left(p_{i}\right)$$

If all possible values of the random variable X are uniformly distributed, then the entropy value increases monotonically. And as the number of possible values n increases, the entropy value also increases. Therefore, in complex network analysis, entropy can be used to identify nodes that are highly connected and relatively uniformly distributed [20].

## 3. Materials and Methods

### 3.1. Algorithmic Information Content and Causality in Network Analysis

When analyzing causality in complex networks, the algorithmic information content proposed by Zenil et al. has become an important tool for understanding causality [5]. It can overcome the limitations of traditional statistical models and reveal the causal relationships of complex networked systems without the need for nonlinear system equations by focusing on the algorithmic information content of the network. The advantage of this approach is that it does not rely on probability distributions or statistical laws to identify causality but rather evaluates the impact on network dynamics by analyzing the algorithmic information complexity of the generating mechanism. Causal mechanisms can also be identified from structures that may appear statistically disordered or random. Traditional statistical methods are mainly used to measure uncertainty and randomness in a system, which cannot fully reveal the causal relationships in a network [28]. In contrast, algorithmic complexity captures additional factors that traditional statistical methods cannot account for, offering a more precise means of assessing causality and providing deeper insights into the causal mechanisms and dynamic behaviors within the network.

### 3.2. Association and Difference Between the MNNGE Method and Causality in Complex Networks

In the field of complex networks, an in-depth understanding of the structure, function, and relationships between nodes is essential. Causality focuses on revealing the causal structure between variables in a network, particularly how changes in the causal connections between nodes affect the entire network structure. On the other hand, the MNNGE method considers multiple interactions among nodes in depth and provides a new perspective for understanding network structure and function by aggregating the gravitational effects of multiple layers of neighbors. These two methods evaluate the relationships between nodes in different ways and contribute to a more comprehensive understanding of the network’s structure and function. However, the two approaches differ in their problem-solving focus, realization principles and approaches, and application areas. The core of causality is to reveal the causal structure between variables in the network, and this analysis is based on algorithmic information theory and causal algorithms to understand the system’s generative mechanisms and dynamics [29]. The core of the MNNGE method focuses on the topology and interaction characteristics of nodes, considering both the overall and local network structures. The MNNGE method measures the interaction relationship between nodes by deeply analyzing their connectivity, location structure, and neighbor attributes to accurately assess the key nodes in complex networks.

### 3.3. The MNNGE Method

The traditional degree centrality [6] approach evaluates a node’s propagation capability by counting the number of its neighbors, and nodes with higher degrees are usually assumed to have stronger propagation capabilities. However, this method overlooks nodes with fewer connections that are located at the core of the network, which may still play a crucial role in information dissemination despite their lower degree. To address this limitation, this paper introduces the K-shell method to reveal the relative positions of nodes in the network. Based on this, this paper defines Node Weight (NW), which combines both the degree and K-shell attributes of a node to measure its initial weight. It is defined as

(3) $$NW\left(i\right)=d\left(i\right)+ks(i)$$

where $d\left(i\right)$ denotes the degree value of node $v_{i}$ and $ks\left(i\right)$ denotes the K-shell value of the node $v_{i}$.

**Figure 1:**
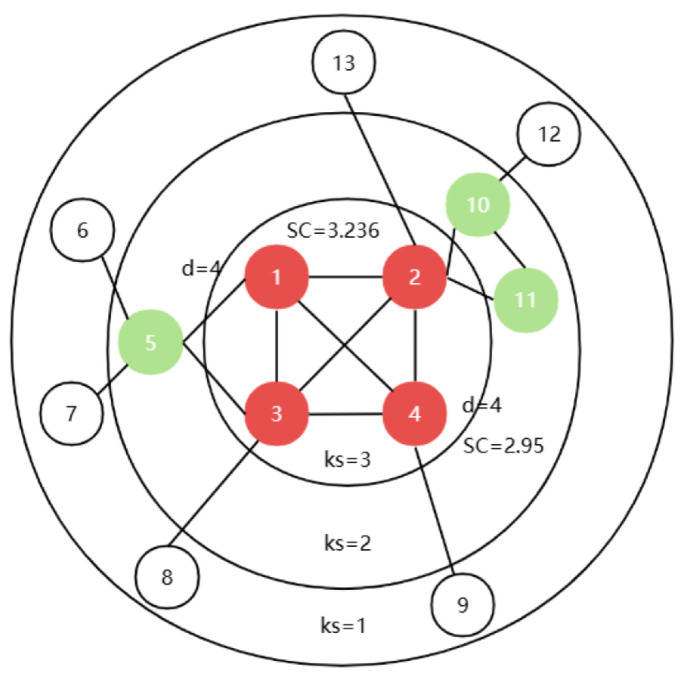
Schematic network.

However, the weights of the nodes are mainly concerned with the number of connections and the network location of the nodes themselves, and further consideration needs to be given to the effect of the interactions between neighboring nodes on the propagation of information. As shown in Figure 1, the degree and K-shell values of node one and node four are 4 and 3, respectively, and their weights are both 7. However, the propagation ability of node one is higher than that of node four. If we continue to analyze the situation of the node’s neighboring nodes, we can get that node one ′s neighboring weights sum to 30, with the distribution of neighboring weights as {9, 8, 7, 6}, and node four ′s neighboring weights are 26, with the distribution of neighboring weights as {7, 9, 8, 2}. It can be concluded that a node has higher propagation ability if it has more neighbors with high weights and uniform weight distribution.

Therefore, this paper proposes Relative Gravitation (RG) as a metric to measure the attractiveness of a node within its neighborhood based on its weight. RG focuses on the node’s position within its neighborhood structure and reflects its attractiveness by evaluating the proportion of the node’s weight relative to the total weight of its neighboring nodes. It is defined as

(4) $$RG\left(i\right)=\frac{NW\left(i\right)}{\sum_{v_{j}\in \Gamma_{i}}NW\left(j\right)}$$

where $\Gamma_{i}$ denotes the set of neighbor nodes of node *i*.

In order to further consider the direct interaction between a node and its neighbors, this paper proposes a new metric called Direct Gravitation (DG). DG reflects the strength of connections between a node and its neighboring nodes by measuring the number of triangular structures formed among them. A triangular structure is a closed subgraph formed by the interconnection of nodes and their neighbors. The more triangular structures a node forms with its neighbors, the tighter the connection between them and the stronger the node’s ability to facilitate the flow of information. To enhance the accuracy of calculating direct gravitation between nodes, this paper not only considers the number of triangular structures between a node and its neighbors but also incorporates the degree information of both the node and its neighbors, thus precisely quantifying the interactive influence between them. It is defined as

(5) $$DG\left(i\right)=\sum_{v_{j}\in \Gamma_{i}}\frac{\left|\Gamma \left(i\right)\cap \Gamma \left(j\right)\right|+1}{d\left(i\right)*d\left(j\right)}$$

where $\left|\Gamma \left(i\right)\cap \Gamma \left(j\right)\right|$ denotes the number of triangular structures between nodes *i* and *j*. The +1 in the formula is to avoid the case of ‘0’ affecting the subsequent entropy calculation.

The combination of relative and direct gravitation not only evaluates the relative importance of a node among its neighbors but also deeply analyzes the local interactions between nodes and their neighbors. To further integrate these two gravitational forces, this paper introduces the method of information entropy to aggregate the relative and direct gravitational forces of multilayer neighbor nodes. The Entropy Centrality (EC) of a node is defined as

(6) $$EC\left(v_{i}\right)=E1\left(v_{i}\right)+E2\left(v_{i}\right)$$

where $E1\left(v_{i}\right)$ and $E2\left(v_{i}\right)$ denote the aggregation of relative and direct gravity of node $v_{i}$, respectively

(7) $$E1\left(v_{i}\right)=-\sum_{v_{j}\in \Gamma_{i}}RG(j)*logRG\left(j\right)$$

(8) $$E2\left(v_{i}\right)=-\sum_{v_{j}\in \Gamma_{i}}DG(j)*logDG\left(j\right)$$

Finally, in order to comprehensively assess the node’s ability to propagate information through multilayer neighbor nodes in the network, this paper proposes Multilayer Neighbor Expansion Centrality (MNEC). MNEC calculates the sum of the entropy centrality of all the neighboring nodes of node $v_{i}$ to measure its influencing ability and extends it to the second-order neighborhood to quantify the propagation potential of a node at different levels in the network. It is defined as

(9) $$MNEC\left(v_{i}\right)=\sum_{v_{j}\in \Gamma_{i}}EC\left(v_{j}\right)$$

The MNNGE value of node *v_i_* is finally calculated by Equation (10)

(10) $$MNNGE\left(v_{i}\right)=\sum_{v_{j}\in \Gamma_{i}}MNEC\left(v_{j}\right)$$

Algorithm 1 shows the pseudocode of the proposed method, which includes detailed information about the computational steps. Lines 2 to 3 calculate the degree and K-shell value of each node with a time complexity of O(|V|). Lines 4 to 10 compute the relative and direct gravitational forces between nodes with a time complexity of O(|V|·⟨k^2^⟩). Lines 11 to 17 aggregate the relative and direct gravitational forces between nodes using entropy, also with a time complexity of O(|V|·⟨k^2^⟩). Lines 18 to 23 and 24 to 29 compute the entropy centrality of the multilayer neighbor extension, both having a time complexity of O(|V|·⟨k^2^⟩). Therefore, the overall time complexity of the MNNGE method is O(|V|·⟨k^2^⟩), where 〈k〉 is the average degree of the network and 〈k^2^〉 is the second-order average degree, and it is much smaller than the total number of its nodes in large-scale networks and can be ignored, which shows that the computational efficiency of the MNNGE is high and applicable to large scale networks.
**Algorithm 1** MNNGE**Input**: G = (V,E)**Output**: A ranking list of the nodes’ influence 1: **Begin** 2: Calculate the degree(v_i_) of each v_i_ ∈ V 3: Assign Ks(v_i_) to each v_i_ ∈ V using K-shell decomposition 4: **for** each node i in G = (V, E) **do** 5:      **for** each node j in G.neighbors of node i **do** 6:            compute the node weight using Equation (3) 7:            compute the relative gravitation using Equation (4) 8:            compute the direct gravitation using Equation (5) 9:      **end for** 10: **end for** 11: **for** each node i in G = (V, E) **do** 12:      **for** each node j in G.neighbors of node i **do** 13:            compute the entropy centrality of E1 using Equation (7) 14:            compute the entropy centrality of E2 using Equation (8) 15:      **end for** 16:      compute the entropy centrality using Equation (6) 17: **end for** 18: **for** each node i in G = (V, E) **do** 19:      Set EC = 0 20:      **for** each node j in G.neighbors of node i **do** 21:            compute the multilayer neighbor expansion centrality using Equation (9) 22:       **end for** 23: **end for** 24: **for** each node i in G = (V, E) **do** 25:      Set MNIEC = 0 26:      **for** each node j in G.neighbors of node i **do** 27:            compute the final information of nodes MNNGE using Equation (10) 28:       **end for** 29: **end for**

To summarize, using node 1 in Figure 1 as an example, the calculation process of the MNNGE method is introduced as follows: First, the weight of node one is calculated using Equation (3) as $NW\left(1\right)=4+3=7$. Then, the relative gravitational force of node one is calculated using Equation (4) as $RG\left(1\right)=\frac{7}{9+8+7+6}=0.233$. Similarly, the relative gravitational force of its neighboring nodes can be calculated as $RG\left(2\right)=0.2727$, $RG\left(3\right)=0.2581$, $RG\left(4\right)=0.2692$, $RG\left(5\right)=0.3158$. To calculate the direct gravitational force of the node, one needs to consider the number of triangles formed by the node and its neighboring nodes. Figure 1 shows that the number of triangles formed by node one and its neighboring nodes {2, 3, 4, 5} are {2, 3, 2, 1}, respectively. Using Equation (5), the direct gravitational force between node one and its neighboring nodes can be calculated through the triangular structure as $DG\left(1\right)=\frac{2+1}{4*6}+\frac{3+1}{4*5}+\frac{2+1}{4*4}+\frac{1+1}{4*4}=0.6375$. Similarly, the direct gravitational force for its neighboring nodes can be calculated as $DG\left(2\right)=0.7944$, $DG\left(3\right)=0.75$, $DG\left(4\right)=0.7125$, $DG\left(5\right)=0.725$. Next, the relative gravitational aggregation of node one is calculated using information entropy through Equation (7) as *E*1(*v*_1_) = $-\left(0.2727*\;log(0.2727)+0.2581*\;log(0.2581)+0.2692*\;log(0.2692)+0.3158*\;log(0.3158)\right)$ = 1.4212. Similarly, its direct gravitational aggregation is $E2\left(v_{1}\right)=0.8732$. The final MNNGE value of node one is calculated as $MNNGE\left(1\right)=39.0710$ using Equation (10).

## 4. Experiments and Results

To validate the accuracy and performance of the MNNGE method for identifying key nodes, DC [6], K-shell [12], CC [7], Cnc+ [13], KSD^w^ [14], KSGC [19], and SHKS [15] were used as baseline methods for comparison with the MNNGE method proposed in this paper.

### 4.1. Datasets

In this paper, experiments were conducted using nine different types of real networks. (1) Karate: a social relationship network between members of the Zachary Karate Club. (2) Lesmis: a network representing the relationships between characters in Victor Hugo’s novel Les Miserables. (3) USAir: a network representing the United States Air Transportation System in 1997. (4) Celegans: the neural connectivity network of the nematode Caenorhabditis elegans. (5) EEC: an email exchange network among members of a major European research institution. (6) Email: a network representing email interactions between users at the University of Rovira i Virgili in Spain. (7) Router: a symmetrized depiction of the Internet’s architecture at the autonomous systems level. (8) Hamsterster: a network of friendship and family relationships among users of the hamsterster.com website. (9) AstroPh: a network of scientific collaborations between authors of papers submitted to the Astrophysics category. These datasets are publicly available and can be downloaded from networkrepository.com and SNAP: Stanford Network Analysis Project.

The information for the nine networks is summarized in Table 1, where N and E denote the number of nodes and edges in the network, ⟨k⟩ is the average degree, ⟨d⟩ denotes the average shortest distance, and ⟨C⟩ is the clustering coefficient of the network. Additionally, *β_th_* and *β* denote the network prevalence threshold and infection probability used in the experiment.

### 4.2. SIR Epidemic Model

To compare the performance of different methods, this paper used the Susceptible-Infected-Recovered (SIR) model to simulate the propagation process of each node in the network [30,31].

In the SIR model, nodes have three states: Susceptible (S), Infected (I), and Recovered (R). The Susceptible (S) state represents a healthy condition in which a node is susceptible to being infected by other nodes. The Infected (I) state indicates that the node is infected and can transmit the infection to susceptible nodes. The Recovered (R) state represents a condition where the node has recovered and can no longer be infected by other nodes.

At the beginning of the propagation process of the SIR model, all nodes in the network are in the susceptible state. During each period, the infected node infects its nearest and next nearest neighbor nodes in the susceptible state with a probability of *β*. Subsequently, each infected node enters the recovery state with a probability of *λ*. This process continues until no nodes remain in the infected state. At the end of the SIR propagation process, the number of recovered nodes is used to indicate the propagation capability of the node. In the SIR propagation process, the value of *β* is related to the epidemic threshold *β_th_* of the network. When *β* is larger than *β_th_*, the disease can spread on a large scale within the network. However, if *β* is smaller, the spread remains limited [20]. Usually, *β* should be slightly larger than the epidemic threshold *β_th_* [13], and the formula for calculating the epidemic threshold *β_th_* is

(11) $$\beta_{th}\approx \frac{\left\langle k\right\rangle }{{\langle k}^{2}\rangle }$$

Where ⟨k⟩ is the average degree of the network, and ${\langle k}^{2}\rangle$ is the second-order average degree. To obtain more accurate experimental data, the number of simulations for the SIR model’s propagation process is determined by the network size: 1000 simulations for small networks with N < 10,000 and 100 simulations for large networks with N ≥ 10,000.

### 4.3. Ranking Accuracy

In this paper, we use the Kendall τ correlation coefficient [32] to evaluate the correlation between different methods and the node importance ranking generated by the SIR model. A higher Kendall τ value indicates a stronger correlation between the method and the SIR ranking results. The Kendall τ is defined as

(12) $$\tau \;\left(R\right)=\frac{2(N_{c}-N_{d})}{N(N-1)}$$

Where *Nc* and $N_{d}$ denote the number of consistent and inconsistent correlations, respectively. Two ranked sequences, $X=\left(x_{1},\;x_{2},\;\cdot \cdot \cdot ,\;x_{n}\;\right)$ and $Y=\left(y_{1},y_{2},\cdot \cdot \cdot ,y_{n}\right)$, are obtained from the SIR model and other different methods. For any pair ($x_{i}$, $y_{i}$) and ($x_{j}$, $y_{j}$), they are consistent if (($x_{i}$ > $x_{j}$) and ($y_{i}$ > $y_{j}$)) or (($x_{i}$ < $x_{j}$) and ($y_{i}$ < $y_{j}$)), and inconsistent otherwise. If $x_{i}$ = $x_{j}$ or $y_{i}$ = $y_{j}$, they are not counted. Kendall’s τ ranges from [−1 to 1], with higher values indicating a stronger agreement between the rankings.

Table 2 demonstrates the Kendall coefficient scores of different methods with SIR ranking results when the network infection rate is *β*. The data clearly show that the MNNGE method is higher than all other methods in terms of Kendall coefficient score, indicating that the MNNGE method is more accurate in identifying key nodes.

In the following experiments, to observe the trend of Kendall τ under different infection probabilities *β*, this paper sets the infection probability *β* as a range from *β_th_* to 2 × *β_th_* during the SIR model propagation simulation. Additionally, the recovery probability *λ* is set to 1 to immunize the node, thereby verifying the accuracy of the MNNGE method in identifying key nodes.

**Figure 2:**
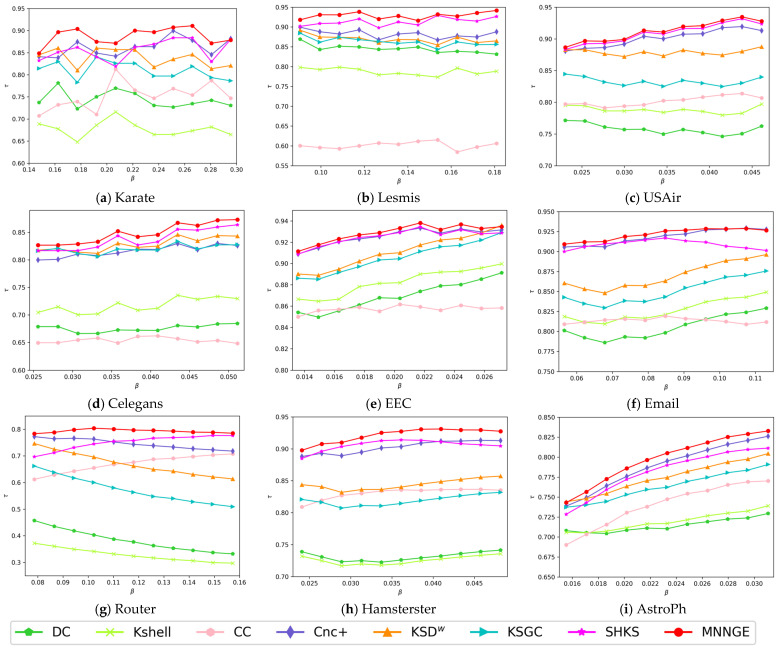
Results of the Kendall τ correlation coefficients of the different methods and the SIR model at different infection probabilities.

Figure 2 shows the variation of Kendall coefficients for the ranking results of different methods compared to the SIR model at various infection rates. It can be observed that the MNNGE method generally shows better performance compared with other methods. Around *β_th_*, the Kendall coefficient values of the MNNGE method are consistently higher than those of other methods. As the value of *β* increases, the method proposed in this paper continues to maintain a lead in terms of accuracy. For example, in the Router, AstroPh, Celegans, Hamsterster, and USAir networks, MNNGE consistently has the highest Kendall τ coefficient. In the Club, EEC, Email, and Lesmis networks, although MNNGE is not the top performer, its τ value remains close to other methods, maintaining a high level of performance. To present the results more clearly, Table 3 shows the average Kendall τ values under different *β*, which further verifies the above conclusion that the MNNGE method proposed in this paper outperforms other methods.

**Figure 3:**
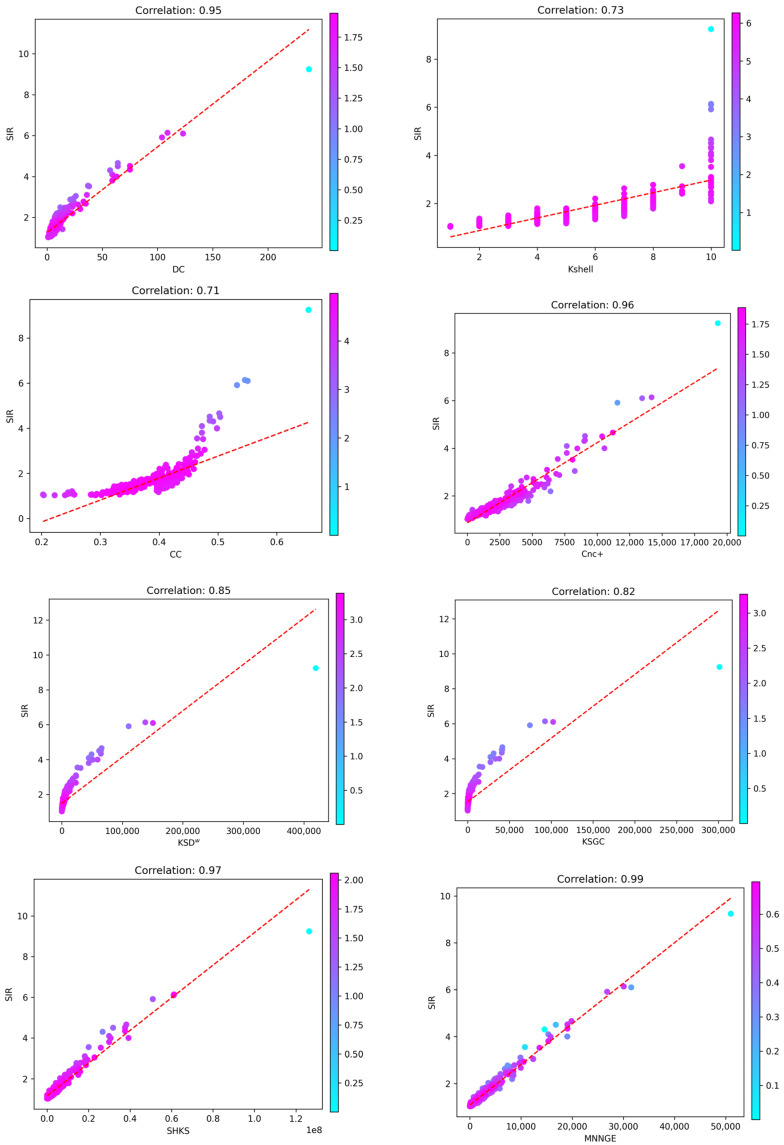
Correlation between the SIR simulation results of the Celegans network and the measurements of different methods.

To further validate the performance of different methods, this paper uses scatter plots to illustrate the correlation between SIR simulation results and the measurements of different methods. Figure 3 shows the correlation between the SIR results and the different methods in the Celegans network. Each point in the graph represents a node in the network, the x-axis represents the node influence measured by different methods, the y-axis represents the real node influence simulated by SIR, and the color of the point indicates the infection ability of each node when the propagation rate *β* = *β_th_*. If the method’s measurements are consistent with the node influence simulated by SIR, all nodes will be distributed on or close to the straight line *y* = *kx* (where *k* > 0). Otherwise, the nodes will be spread out and away from the line.

Figure 3 shows that the CC and K-shell methods have a weaker performance, and their node distributions are more dispersed, indicating a large deviation between their measurements and the real situation of SIR simulation. In contrast, the KSGC and KSD^w^ methods are improved, but their correlation with the SIR simulation results is still weak. The DC and Cnc+ methods perform better, showing a stronger correlation with the SIR results. However, the MNNGE method proposed in this paper, as well as the SHKS method, exhibit higher performance. The node distributions of these two methods are closely centered around *y* = *kx*, indicating that their measurements are highly consistent with the real node influence of SIR simulations, and the MNNGE method exhibits higher correlation compared to SHKS, further indicating that the MNNGE method has higher accuracy and effectiveness in identifying key nodes.

### 4.4. Comparison of Identifying the Top-k Key Nodes

The Kendall correlation coefficient experiment considers the ranking of all the nodes in the network. However, in key node identification, it is more important to find the top-ranked nodes rather than obtaining a ranked list of all nodes. Therefore, in this paper, we use the Jaccard similarity coefficient to assess the similarity of the top-k nodes in two ranked lists [33]. The Jaccard similarity coefficient is defined as

(13) $$J\left(L\right)=\frac{\left|\sigma \left(L\right)\cap R\left(L\right)\right|}{\left|\sigma \left(L\right)\cup R\left(L\right)\right|}$$

where σ(L) and R(L) denote the set of top L nodes in the real ranking table σ and the ranking table R obtained by different methods, respectively.

**Figure 4:**
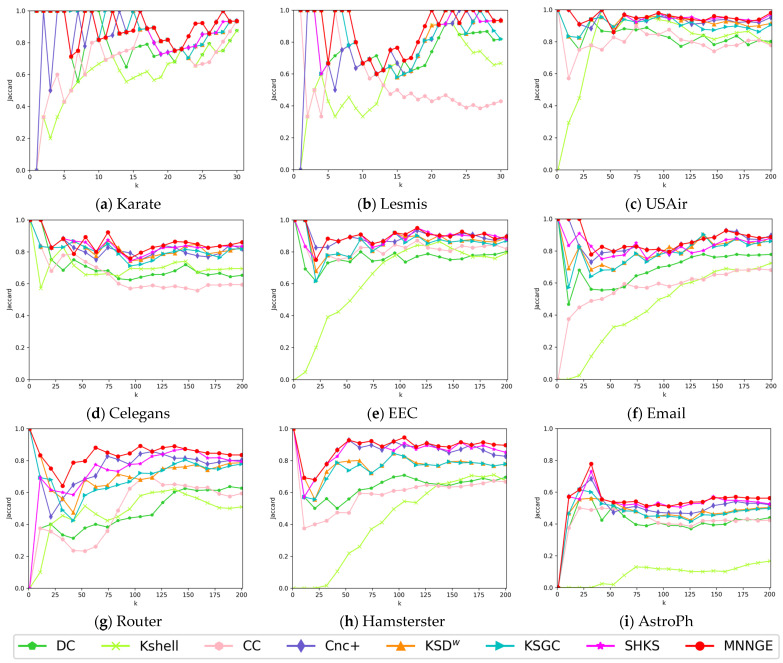
Jaccard similarity coefficients for the top-k key nodes.

Figure 4 shows the Jaccard similarity coefficient results for identifying the top-k key nodes in nine real networks. The results show that the MNNGE method performs best on Routers, Hamsterster, and AstroPh networks. On the EEC, Email, Celegans, Lesmis, and Usair networks, the ranking similarity results remain at a high level, although they may not always be optimal. Even on the Karate network, where MNNGE shows fewer advantages, its performance is close to optimal. These results show that the MNNGE method proposed in this paper is highly accurate in identifying the top-k nodes and exhibits higher performance compared to other methods.

### 4.5. Monotonicity

In this paper, the monotonicity of different methods is evaluated using the ranked monotonicity function [13]. When each node is assigned a unique ranking index, the method is considered to have higher discriminative ability. The differentiation ability of the ranked table generated by different methods is calculated as

(14) $$M\left(R\right)={\left[1-\frac{\sum_{r\in R}N_{r}(N_{r}-1)}{N(N-1)}\right]}^{2}$$

where σ(L) and R(L) denote the set of top L nodes in the real ranking table σ and the ranking table R obtained by different methods, respectively.

$N_{r}$ represents the number of nodes with the same ranking index, *r*, and *N* is the total number of nodes. The value of M(R) ranges between [0 and 1], with a larger M(R) indicating a higher differentiation ability of the method.

Table 4 shows the monotonicity performance of different methods in generating ranked lists. The results demonstrate that both the MNNGE and SHKS methods exhibit strong differentiation abilities. In contrast, the K-shell method shows weaker differentiation, primarily because it struggles to distinguish between nodes with identical K-shell values. The DC method also has a relatively low monotonicity score, as it only takes into account the number of a node’s direct connections while ignoring the node’s global position in the network. On the other hand, the CC method performs better because it integrates the degree of association between a node and its neighbors. It is worth noting that Cnc+, KSD^w^, KSGC, SHKS, and the MNNGE method proposed in this paper not only focus on the node’s own characteristics but also comprehensively take into account the influence of neighboring nodes. As a result, these methods generally exhibit better monotonicity. Especially on the three datasets, Karat, Router, and AstroPh, the MNNGE method shows higher monotonicity, indicating that the method has a stronger differentiation ability.

**Figure 5:**
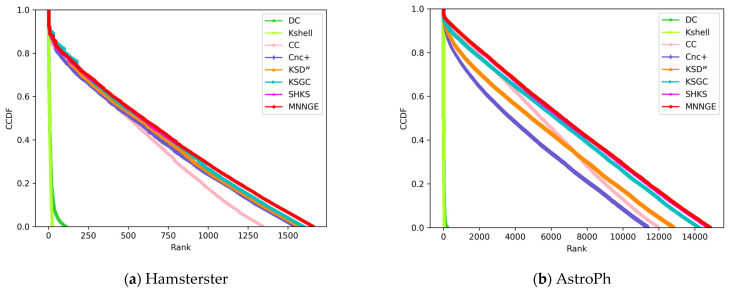
Complementary cumulative distribution of the node influential ranks.

To more clearly illustrate the ranking distributions obtained by different methods, this paper introduces the complementary cumulative distribution function (CCDF) in addition to the monotonicity function to compare the ranking distributions of different methods [34]. The CCDF is defined as F(Y)=P(Y>y), where P(Y>y) denotes the probability that an element in *Y* exceeds a given value y. A slower decrease in the slope of the CCDF indicates that a wider range of ranks is assigned to the nodes, which is indicative of better rank distribution performance. Figure 5 shows the ranking distribution results of different methods on the Hamsterster and AstroPh networks. As shown in the figure, the CCDF values of the DC and K-shell methods decrease more rapidly, indicating that they perform poorly in assigning different ranking values to nodes. Although Cnc+ and CC decrease at a slightly slower rate than them, they are still not sufficiently precise in ranking node values. In contrast, the KSGC, KSD^w^, and SHKS methods show a much smoother decline, implying that they are able to assign ranking values to nodes more accurately. However, the MNNGE method exhibits superior performance to these methods, achieving the best monotonicity and ranking distribution performance among all the compared methods.

## 5. Discussion

In complex networks, accurately identifying key nodes is crucial to control the propagation of information. In this paper, we propose a key node identification method, MNNGE, based on multilayer neighbor node gravity and information entropy. The method comprehensively considers the node’s own attributes and the relative and direct gravity between the node and its neighbors. It aggregates these factors through information entropy and extends them to the second-order neighborhood, thereby improving the accuracy of key node identification. To validate the accuracy and effectiveness of the MNNGE method, this paper conducts multiple sets of experiments on nine real datasets and compares them with DC, K-shell, CC, Cnc+, KSDw, KSGC, and SHKS methods. The experimental results demonstrate that the MNNGE method performs better in terms of the Kendall τ correlation coefficient, top-k node identification, monotonicity, and complementary cumulative distribution function. Moreover, the results obtained by the MNNGE model are highly consistent with the SIR model, indicating that the MNNGE method can accurately identify key nodes in complex networks. Furthermore, the MNNGE method is applicable to large-scale networks without requiring any parameter setting, making it a versatile tool for identifying key nodes. Future research can build upon this foundation to further explore the problem of key node identification in dynamic networks.

## Figures and Tables

**Table 1:** Attributes of the dataset used in the experiment.

Network	V	E	⟨k⟩	⟨d⟩	⟨C⟩	Assortativity	*β_th_*	*β*
Karate	34	78	4.59	2.41	0.57	−0.4756	0.1477	0.15
Lesmis	77	254	6.60	2.64	0.57	−0.1652	0.0905	0.15
USAir	332	2126	12.81	2.74	0.63	−0.2079	0.0231	0.03
Celegans	453	2025	8.94	2.66	0.65	−0.2258	0.0256	0.03
EEC	986	16,064	32.59	2.59	0.41	−0.0257	0.0136	0.015
Email	1133	5451	9.62	3.61	0.22	0.0782	0.0565	0.1
Router	5022	6258	2.49	6.45	0.011	−0.1384	0.0786	0.1
Hamsterster	2426	16,631	13.71	3.67	0.54	0.0474	0.0241	0.03
AstroPh	18,772	198,050	21.102	4.19	0.63	0.2051	0.015	0.02

**Table 2:** Kendall τ correlation results between the different methods and the SIR model at the infection probability *β*.

Network	τ (DC)	τ (K-Shell)	τ (CC)	τ (CNc+)	τ (KSD^w^)	τ (KSGC)	τ (SHKS)	τ (MNNGE)
Karate	0.7023	0.5829	0.7736	0.8093	0.8414	0.7879	0.8164	0.8734
Lesmis	0.8028	0.7273	0.5971	0.8735	0.8612	0.8489	0.9146	0.9282
USAir	0.7183	0.7387	0.8027	0.8966	0.8699	0.8223	0.9030	0.9066
Celegans	0.6352	0.6492	0.6641	0.8124	0.8300	0.8199	0.8241	0.8409
EEC	0.8446	0.8503	0.8610	0.9186	0.8992	0.8900	0.9175	0.9212
Email	0.7944	0.7983	0.8115	0.9287	0.8850	0.8645	0.9082	0.9315
Router	0.2964	0.1749	0.6520	0.7558	0.6871	0.5938	0.7379	0.7973
Hamsterster	0.7059	0.6963	0.8261	0.8918	0.8342	0.8084	0.9059	0.9139
AstroPh	0.6933	0.6940	0.7255	0.7727	0.7612	0.7506	0.7675	0.7812

**Table 3:** Mean Kendall τ correlation results between the different methods and SIR.

Network	τ (DC)	τ (K-Shell)	τ (CC)	τ (CNc+)	τ (KSD^w^)	τ (KSGC)	τ (SHKS)
Karate	0.7440	0.6775	0.7519	0.8616	0.8385	0.8102	0.8558
Lesmis	0.8448	0.7876	0.6014	0.8823	0.8833	0.8627	0.9132
USAir	0.7577	0.7881	0.8021	0.9012	0.8789	0.8328	0.9088
Celegans	0.6758	0.7174	0.6540	0.8157	0.8280	0.8198	0.8372
EEC	0.8695	0.8827	0.8573	0.9253	0.9111	0.9057	0.9249
Email	0.8056	0.8267	0.8134	0.9183	0.8702	0.8505	0.9087
Router	0.3825	0.3281	0.6699	0.7455	0.6703	0.5728	0.7505
Hamsterster	0.7313	0.7258	0.8304	0.9026	0.8443	0.8192	0.9057
AstroPh	0.7144	0.7194	0.7402	0.7894	0.7746	0.7632	0.7816

**Table 4:** Monotonicity results from different methods.

Network	M (DC)	M (K-Shell)	M (CC)	M (CNc+)	M (KSD^w^)	M (KSGC)	M (SHKS)	M (MNNGE)
Karate	0.7079	0.4958	0.8993	0.94724	0.9542	0.9542	0.9542	0.9612
Lesmis	0.8147	0.7441	0.9414	0.9580	0.9580	0.9580	0.9580	0.9580
USAir	0.8586	0.8114	0.9892	0.9945	0.9942	0.9943	0.9951	0.9951
Celegans	0.7922	0.6962	0.9899	0.9975	0.9971	0.9970	0.9987	0.9987
EEC	0.9571	0.9216	0.9983	0.9998	0.9998	0.9999	0.9999	0.9999
Email	0.8874	0.8088	0.9988	0.9991	0.9993	0.9995	0.9999	0.9999
Router	0.2886	0.0691	0.9961	0.9660	0.9393	0.9399	0.9964	0.9965
Hamsterster	0.8980	0.8714	0.9851	0.9855	0.9854	0.9854	0.9857	0.9857
AstroPh	0.9264	0.9083	0.9990	0.9989	0.9990	0.9990	0.9991	0.9992

## Data Availability

The datasets generated and analyzed during the current study are available at The Network Data Repository (networkrepository.com) and SNAP (SNAP: Stanford Network Analysis Project).
